# Precise definition of PTEN C-terminal epitopes and its implications in clinical oncology

**DOI:** 10.1038/s41698-019-0083-4

**Published:** 2019-04-15

**Authors:** Janire Mingo, Sandra Luna, Ayman Gaafar, Caroline E. Nunes-Xavier, Leire Torices, Lorena Mosteiro, Rebeca Ruiz, Isabel Guerra, Roberto Llarena, Javier C. Angulo, José I. López, Rafael Pulido

**Affiliations:** 1 Biocruces-Bizkaia Health Research Institute, Barakaldo, Spain; 2 Department of Pathology, Cruces University Hospital, Barakaldo, Spain; 3 Department of Tumor Biology, Institute for Cancer Research, Oslo University Hospital Radiumhospitalet, Oslo, Norway; 4 Department of Pathology, Araba University Hospital, Vitoria, Spain; 5 University of the Basque Country, Leioa, Spain; 6 Department of Urology, Cruces University Hospital, Barakaldo, Spain; 7 Department of Urology, University Hospital of Getafe, Getafe, Madrid, Spain; 8 Clinical Department, European University of Madrid, Laureate Universities, Madrid, Spain; 9 Ikerbasque, Basque Foundation for Science, Bilbao, Spain

## Abstract

Anti-PTEN monoclonal antibodies (mAb) are arising as important tools for immunohistochemistry (IHC) and protein quantification routine analysis in clinical oncology. Although an effort has been made to document the reliability of tumor tissue section immunostaining by anti-PTEN mAb, and to standardize their IHC use in research and in the clinical practice, the precise topological and biochemical definition of the epitope recognized by each mAb has been conventionally overlooked. In this study, six commercial anti-PTEN mAb have been validated and characterized for sensitivity and specificity by IHC and FISH, using a set of prostate and urothelial bladder tumor specimens, and by immunoblot, using PTEN positive and PTEN negative human cell lines. Immunoblot precise epitope mapping, performed using recombinant PTEN variants and mutations, revealed that all mAb recognized linear epitopes of 6–11 amino acid length at the PTEN C-terminus. Tumor-associated or disease-associated mutations at the PTEN C-terminus did not affect subcellular localization or PIP3 phosphatase activity of PTEN in cells, although resulted in specific loss of reactivity for some mAb. Furthermore, specific mimicking-phosphorylation mutations at the PTEN C-terminal region also abolished binding of specific mAb. Our study adds new evidence on the relevance of a precise epitope mapping in the validation of anti-PTEN mAb for their use in the clinics. This will be substantial to provide a more accurate diagnosis in clinical oncology based on PTEN protein expression in tumors and biological fluids.

## Introduction

Expression of biomarkers, as detected by standard immunohistochemistry (IHC), together with classic histological parameters, constitutes the first-line of diagnosis of most solid tumors.^1,2^ IHC has also arisen as a universal prognostic technique to assist in patient stratification and therapy decisions in oncology.^3,4^ It is currently admitted that sensitivity, specificity, and reproducibility are essential factors to validate monoclonal antibodies (mAb) as IHC tools in research and in the clinical practice.^5–8^ However, unless short defined synthetic peptides are used as immunogens in the mAb obtention, the precise topological and biochemical definition of the epitope recognized by the mAb is mostly overlooked. This may be informative to define potential antigen cross-reactivities, and may provide additional information to generate improved rational-design mAb.^9,10^ More importantly, precise epitope mapping is relevant when the biosynthesis of the marker protein is influenced by mRNA alternative splicing or alternative translation mechanisms, or when the mature marker protein is targeted by dynamic post-translational modifications, such as phosphorylation.^11^ In addition, many IHC marker proteins are frequently targeted for mutations in tumors, which could affect in several ways both the protein function and its recognition by specific mAb, with important prognostic implications.^12,13^

The PTEN protein has emerged as one of the most important tumor suppressors in human cancer, with a high potential as prognostic and prediction-of-response marker in several human cancers, including those with high prevalence such as breast or prostate cancer.^14–18^ PTEN exerts its tumor suppressor functions mainly through the negative regulation of the activity of the PI3K/AKT pro-survival pathway, by dephosphorylating at cell membranes the PI(3,4,5)P3 (PIP3) reaction product of the oncogenic PI3K. In addition, PTEN plays PIP3-independent tumor suppressor roles in the cytoplasm and in the nucleus, and the dynamic partitioning of PTEN between membranes, cytoplasm, and nucleus, is crucial in modulating PTEN physiologic activity.^19–22^ The *PTEN* gene behaves as a haploinsufficient gene, and partial loss of expression or activity of PTEN protein confers tumor growth advantages. As a consequence, the *PTEN* gene is frequently targeted by deletions and mutations in tumors, and heterozygous *PTEN* mutations are found in the germline from patients with hamartomas and tumor predisposition (PHTS).^16,23–25^ Aberrant alterations in PTEN cytoplasmic/nuclear localization have also been found in tumors and in the germline of PHTS patients,^26–32^ making important for precise diagnosis not only the detection of the protein in tumor samples, but also its location in the cell. In this regard, recent studies have unveiled the existence of alternatively translated PTEN isoforms, including PTEN-L, which possesses a variable N-terminal extension that targets the protein for secretion and to different cell compartments.^33–35^ In addition, several PTEN splice variants have been identified,^36,37^ among which PTEN-Δ, lacking the C-terminal PTEN residues encoded in exon 9, has been proposed to have similar function as PTEN.^38^

PTEN protein is composed of two well-ordered structural domains, a protein tyrosine phosphatase (PTP) catalytic N-terminal domain (residues 8–185) and a membrane-binding C2 C-terminal domain (residues 186–352). In addition, PTEN possesses several intrinsically disordered protein regions (IDPRs), including a short N-terminal segment (residues 1–7), a regulatory C-terminal tail (residues 353–403), and an internal loop (residues 286–309) at the C2 domain.^39^ The N-terminal extension of PTEN-L (residues 1-L to 173-L), which is not present in the more abundant canonical PTEN, is also an IDPR.^40^ The PTEN C-terminal tail is targeted by post-translational modifications, including phosphorylation, acetylation, and caspase-3 cleavage,^41–44^ and plays a major role in PTEN function by mediating inter- and intra-molecular protein–protein interactions that regulate PTEN stability, subcellular localization, and catalysis.^45–50^ Deletion of the PTEN C-terminal tail generates a relatively unstable protein, which is enriched in membranes and in the nucleus.^51–54^ Further truncations of PTEN into the C2 domain are deleterious for PTEN protein stability and function,^55^ highlighting the pathological importance of most of the premature termination codon mutations targeting the *PTEN* gene.

Anti-PTEN mAb suitable to detect by IHC the expression of PTEN in tumor tissues were generated and described soon after the discovery of PTEN as a major tumor suppressor.^56,57^ Significant efforts have been made since then to technically optimize, validate, and standardize the available anti-PTEN mAb for their IHC reliable use in research and diagnosis.^58–70^ However, precision studies aiming to define the topological and molecular properties of the epitopes recognized by these anti-PTEN mAb, which is substantial to understand their immunostaining patterns, are still lacking. Here, we have performed a sensitivity-validation and specificity-validation analysis, and a precision epitope mapping of six commercially available anti-PTEN mAb suitable for IHC techniques. Our analysis has unveiled a major immunodominant role for the distal PTEN disordered C-terminal tail, where linear epitopes of 6–11 residues length were located. In addition, our findings illustrate how post-translationally modified PTEN forms, or specific PTEN variants associated to disease, may display altered recognition by specific anti-PTEN mAb, which could have important diagnostic implications.

## Results

### Specificity and sensitivity of anti-PTEN mAb

**Fig. 1:**
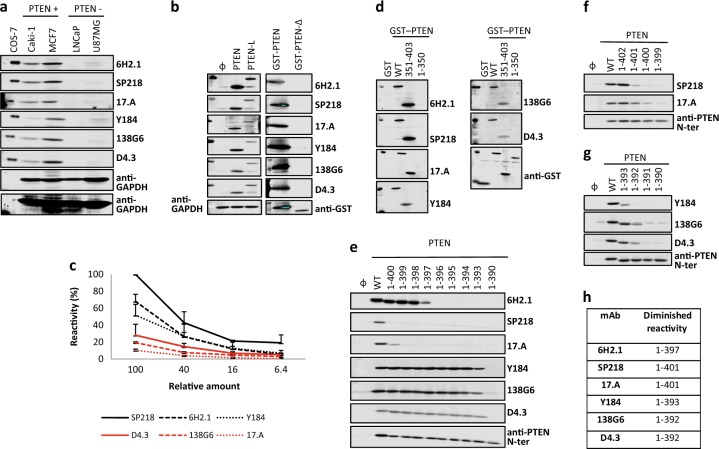
Specificity, sensitivity, and reactivity of anti-PTEN mAb with PTEN C-terminus. **a** Specificity of anti-PTEN mAb. Detection of endogenous PTEN protein by the different anti-PTEN mAb by immunoblot, using cell lysates from PTEN-positive (Caki-1 and MCF7) and PTEN-negative (LNCaP and U87MG) cell lines. Low-exposure and high-exposure images of anti-GAPDH blots are shown as a control. Detection of recombinant PTEN 1–403, from lysates from transfected COS-7 cells, is also shown. **b** Recognition of PTEN isoforms. In the left panel, detection of recombinant PTEN 1–403 and PTEN-L 1-L-576-L by immunoblot from lysates from transfected COS-7 cells is shown, and detection of GAPDH with anti-GAPDH antibody is shown as a control. In the right panel, detection of recombinant GST-PTEN 1-403 and GST-PTEN-Δ 1-343-Ser is shown, and detection using anti-GST antibody is shown as a control. Ø, empty vector. **c** Sensitivity of anti-PTEN mAb. Reactivity of the anti-PTEN mAb with decreasing amounts of recombinant PTEN from cell lysates from transfected COS-7 cells. Data are shown as relative mAb reactivity by immunoblot (mean ± s.d. from three independent experiments), as determined by PTEN protein band quantification. **d** Reactivity of anti-PTEN mAb with PTEN C-terminus. Detection of GST PTEN 1–403 (WT), GST PTEN 351–403, and GST PTEN 1–350 by the different anti-PTEN mAb by immunoblot, as in **b**. Detection using anti-GST antibody is also shown as a control. GST, GST alone. **e**–**g** Reactivity of anti-PTEN mAb with PTEN C-terminus. Detection of PTEN C-terminal truncations, as in **b**. Detection using a polyclonal antibody recognizing PTEN N-terminus (anti-PTEN N-ter) is also shown as a control. WT, PTEN 1-403; Ø, empty vector. **h** Summary of the diminished reactivity of the different anti-PTEN mAb with PTEN C-terminal truncations

Six commercial anti-PTEN mAb (6H2.1, SP218, 17.A, Y184, 138G6, and D4.3) suitable for IHC were included in our study (Table 1). Specificity was assessed by immunoblot using PTEN-positive (Caki-1, MCF7) and PTEN-negative (LNCaP, U87MG) human cancer cell lines. As shown, all mAb recognized specifically endogenous PTEN in the PTEN-positive cell lines (Fig. 1a). In addition, all mAb recognized by immunoblot recombinant PTEN (residues 1–403) and PTEN-L translational isoform (residues 1-L-576-L) overexpressed in COS-7 cells. However, none of the mAb did react with a recombinant form of the PTEN-Δ splice variant (residues 1–343 followed by an additional Ser residue) (Fig. 1b). IHC specificity of the anti-PTEN mAb was assessed on a panel of 81 FFPE prostate adenocarcinoma samples, in comparison with FISH analysis using a *PTEN* gene-specific probe (Table 2). In addition, a panel of 49 FFPE urothelial bladder carcinoma samples was also analyzed (Table S1). As shown, the 6H2.1 and SP218 mAb gave the best IHC specificity scores in the prostate samples, without false positives when correlated with the absence of *PTEN* gene by FISH analysis (Table 2; Fig. S1). Negative immunostaining was detected with all anti-PTEN mAb in a variable number of prostate or urothelial bladder samples positive for FISH analysis (Table 2, Table S1), suggesting the frequent loss of PTEN protein expression in these tumor types without deletion of the *PTEN* gene, in agreement with previous observations by others.^17,71,72^ Sensitivity of the anti-PTEN mAb was tested by immunoblot using decreasing amounts of cell lysates containing ectopically expressed recombinant PTEN from transfected COS-7 cells. In these assays, the SP218, 6H2.1, and Y184A mAb displayed the higher sensitivity to detect PTEN (Fig. 1c).

**Table 1:** Characteristics of the mAb used in this study

mAb	Isotype	Host	Immunogen^a^	Reference^b^
6H2.1	IgG	Mouse	PTEN 304-403	^56^
SP218	IgG	Rabbit	C-terminal synthetic PTEN peptide	^60^
17.A (Ab-4)	IgM	Mouse	PTEN 2-403	^57^
Y184	IgG	Rabbit	C-terminal synthetic PTEN peptide	^70^
138G6	IgG	Rabbit	C-terminal synthetic PTEN peptide	^81^
D4.3		Rabbit	C-terminal synthetic PTEN peptide	^82^

^a^Amino acid numbering is indicated, according to NP_000305.3. No information is available on the amino acid sequence of the synthetic peptides used as immunogens

^b^The reference where the mAb was first described (to the best of our knowledge) is indicated

**Table 2:** Comparative IHC and FISH analysis of anti-PTEN mAb using a panel of FFPE prostate carcinomas

			*FISH*− .		*FISH*+ .		*FISH*++^a^.	
mAb	neg/pos^b^	%neg	neg	pos	neg	pos	neg	pos
6H2.1	62/19	76.5	8	0	12	4	42	15
SP218	50/31	61.7	8	0	11	5	31	26
17.A	41/40	49.4	3	5	7	9	31	26
Y184	33/48	40.7	5	3	4	12	24	33
138G6	45/36	55.6	7	1	9	7	29	28
D4.3	42/39	51.8	6	2	8	8	27	30

^a^No PTEN signal; +, 1 PTEN signal; ++, 2 PTEN signals

^b^Number of negative/positive samples for IHC staining

### Definition of the epitopes recognized by anti-PTEN mAb at the PTEN intrinsically disordered C-terminal tail

**Fig. 2:**
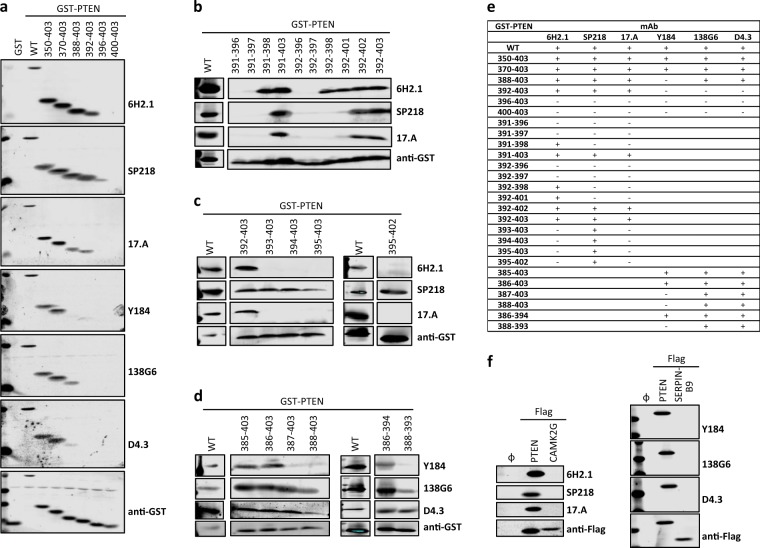
Reactivity of anti-PTEN mAb with peptides from PTEN C-terminal tail and with potential cross-reacting proteins. **a**–**d** Detection of recombinant GST-fusion proteins containing peptides from the PTEN C-terminal tail, as in Fig. 1. Detection using anti-GST antibody is also shown as a control. WT, GST-PTEN 1–403; GST, GST alone. **e** Summary of the reactivity of the different anti-PTEN mAb with the GST-fusion proteins containing peptides from the PTEN C-terminal tail. +, binding; −, no binding. **f** Reactivity of the anti-PTEN mAb with SERPINB9-Flag or CAMK2G-Flag. Detection using anti-Flag antibody is shown as a control. Ø, empty vector

As shown in Table 1, most of the immunogens used to obtain the anti-PTEN mAb under study are C-terminal PTEN fragments or peptides, which is consistent with the lack of recognition by these mAb of the PTEN-Δ splice variant (Fig. 1b). Furthermore, all the mAb recognized by immunoblot a recombinant GST-PTEN fusion protein encompassing the PTEN C-terminal tail (PTEN 351–403), but not a GST-PTEN protein lacking this region (PTEN 1–350) (Fig. 1d). This demonstrates that the epitopes recognized by the distinct anti-PTEN mAb reside at the 350–403 PTEN C-terminal IDPR, and that the rest of PTEN protein is dispensable for mAb recognition. To ascertain more precisely the minimal PTEN region recognized by the distinct mAb, sequential deletion of individual C-terminal PTEN residues was performed, and mAb reactivity was monitored (Fig. 1e–g). Interestingly, this analysis disclosed several differential patterns of anti-PTEN mAb reactivity, which are summarized in Fig. 1h. Whereas the SP218 mAb was the more sensitive to PTEN C-terminal deletion (diminished reactivity with the PTEN 1–401 deletion), the 138G6 and D4.3 mAb were the more resistant to PTEN C-terminal deletion (diminished reactivity with the PTEN 1-392 deletion). These results suggest that the anti-PTEN mAb analyzed recognize distinct linear epitopes at the PTEN C-terminus. To delimit the minimal PTEN region recognized by the mAb, we tested their reactivity with GST-PTEN fusion proteins with an intact C-terminus but progressive N-terminal deletions. As shown in Fig. 2a and summarized in Fig. 2e, a GST-PTEN 370-403 protein was recognized by all mAb, while a GST-PTEN 396-403 protein was not recognized by any of the mAb. Deletion of PTEN residues in the region 370-396 distinguished the reactivity of two groups of mAb: 6H.2, SP218, and 17.A mAb, recognizing a more C-terminal epitope; and Y184, 138G6, and D4.3 mAb, recognizing a more N-terminal epitope (Fig. 2a, b). Finally, additional PTEN-deletion analysis was performed separately on the region recognized by these two groups of mAb (Fig. 2c, d), and a summary of the results is shown in Fig. 2e. Next, we performed an in silico search for potential human antigens cross-reacting with the analyzed anti-PTEN mAb. BLAST sequence homology searches using the human PTEN amino acid sequences 385–395 [(385)SDPENEPFDED(395)] and 391–402 [(391)PFDEDQHTQITK(402)] rendered partial matches with non-PTEN human proteins. The closest matches to the minimal epitopes defined for the anti-PTEN mAb included sequences from SERPINB9 [peptide (389)NEPFDE(394), numbering corresponds to PTEN amino acid sequence] and from CAMK2 isoforms [peptide (393)DEDQH(397)]. However, neither SERPINB9 nor CAMK2G were recognized by immunoblot by the corresponding anti-PTEN mAb (Fig. 2f). Together, our analyses demonstrate that the distinct anti-PTEN mAb studied specifically recognize overlapping but different linear epitopes at the very PTEN C-terminus.

### Phosphorylation-mimicking and tumor-associated mutations at the PTEN C-terminus differentially abrogate anti-PTEN mAb reactivity

**Fig. 3:**
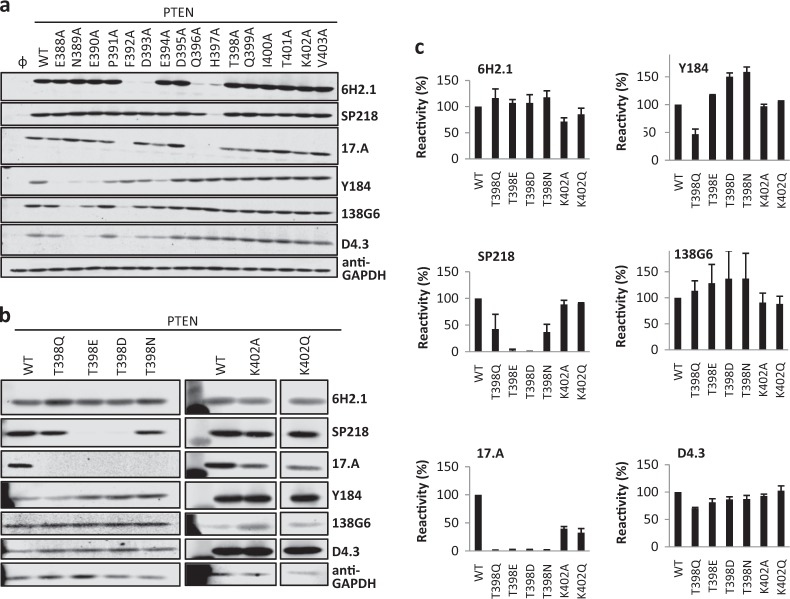
Reactivity of anti-PTEN mAb with PTEN variants containing amino acid substitutions at PTEN C-terminal tail. **a** Reactivity of anti-PTEN mAb with Ala-substitution mutations at PTEN C-terminus, as in Fig. 1. Detection of GAPDH with anti-GAPDH antibody is shown as a control. Ø, empty vector. **b**, **c** Reactivity of anti-PTEN mAb with amino acid substitutions mimicking phosphorylation or acetylation at PTEN C-terminus, as in Fig. 1. Both immunoblot images (**b**) and quantification of the relative mAb reactivity (**c**) (mean ± s.d. from two independent experiments) is shown

The definition of the PTEN residues specifically recognized by each anti-PTEN mAb allowed us to test the possibility that anti-PTEN mAb reactivity could be affected by single amino acid substitutions of these residues. First, we performed an Ala-scanning mutagenesis analysis of the residues configuring the different PTEN C-terminal epitopes. This analysis unveiled different reactivity patterns for the distinct anti-PTEN mAb, and confirmed the differential dependence for mAb recognition on the PTEN C-terminal regions defined in the C-terminal deletion analysis. Furthermore, the Ala-scanning analysis illustrated that the very C-terminal PTEN residues were necessary, but not sufficient, to configure the epitopes recognized by some of the anti-PTEN mAb (Fig. 3a). Next, we tested the effect of physiologic post-translational modifications targeting these residues in the recognition by the different mAb. The Thr398 PTEN residue is phosphorylated by the DNA damage-responsive ATM kinase.^73^ Phosphorylation-mimicking substitution of PTEN Thr398 residue to Glu or Asp (mutations T398E and T398D), but not substitution to Ala, Gln, or Asn (mutations T398A, T398Q, and T398N), specifically abrogated the recognition of PTEN by the SP218 mAb. On the other hand, the reactivity of the 17.A mAb towards the T398Q, T398E, T398D, and T398N mutations was lost, but not towards the T398A mutation. Finally, the reactivity of the other anti-PTEN mAb towards PTEN was not affected by the Thr398 substitutions (Fig. 3b, c). These results suggest that Thr398 phosphorylation could affect negatively the reactivity of SP218 and 17.A mAb towards PTEN. The Lys402 PTEN residue is acetylated by the CBP acetyltransferase, which impacts PTEN binding to PDZ domains,^42^ but acetylation-mimicking substitution of Lys402 to Gln (mutation K402Q) did not affect the recognition of PTEN by any of the mAb (Fig. 3b, c).

**Fig. 4:**
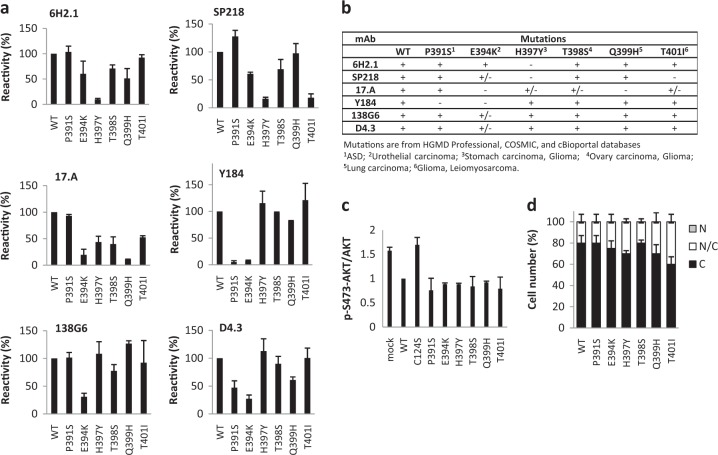
Recognition by anti-PTEN mAb and functional properties of disease-associated PTEN C-terminal tail variants. **a** Reactivity of anti-PTEN mAb with amino acid substitutions at PTEN C-terminus found in tumors or in patients. Quantification of the reactivity is shown (mean ± s.d. from two independent experiments), as in Fig. 3c. **b** Summary of the reactivity of the different anti-PTEN mAb with amino acid substitutions found in tumors or in patients mutations. +, binding; +/− diminished binding; -, no binding. **c** Functional activity of the disease-associated PTEN variants. Data are shown as the ratio pSer473-AKT/AKT (mean ± s.d. from two independent experiments), quantified by immunoblot with anti-pSer473-AKT and anti-AKT antibodies, from lysates from COS-7 cells co-transfected with PTEN and AKT1. **d** Subcellular localization of disease-associated PTEN variants. Data are shown as the percentage of cells displaying cytoplasmic (C), nuclear/cytoplasmic (N/C), or nuclear (N) localization (mean ± s.d. from two independent experiments)

Next, reactivity of the anti-PTEN mAb with tumor-associated or disease-associated PTEN variants targeting residues at the PTEN 391–403 region was tested. As shown in Fig. 4a and summarized in Fig. 4b, the reactivity of specific anti-PTEN mAb with selective PTEN variants at this region was lost, in a manner which was consistent with the precise epitope mapping assigned to each mAb. Importantly, functional analysis of these PTEN variants revealed that all the variants displayed PIP3 phosphatase activity in cells and subcellular location equivalent to PTEN wild type, as monitored by phospo-AKT content immunoblot and by immunofluorescence analysis, respectively (Fig. 4c, d, respectively). Together, these results illustrate that the recognition of functional PTEN proteins by specific anti-PTEN mAb may be affected by particular PTEN protein post-translational modifications or *PTEN* gene mutations.

## Discussion

**Fig. 5:**
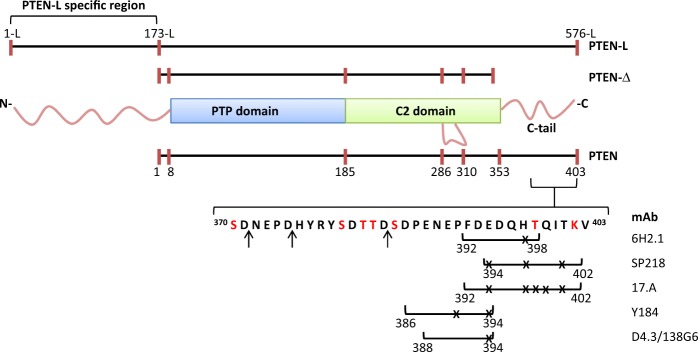
Schematic depiction of PTEN isoforms indicating the amino acid sequences of the linear epitopes recognized by the different anti-PTEN mAb. Numbers indicate the amino acid numbers. Numbering of PTEN-L is according to ref. ^35^ Amino acids are indicated by one-letter code. Ser and Thr residues in red are physiologically phosphorylated residues. The Lys residue in red can undergo acetylation. The arrows indicate the residues where PTEN is cleaved by caspase-3. The lines under the depicted amino acid sequence delimit the epitopes recognized by each mAb. Crosses indicate residues mutated in tumors or in patients whose substitution causes total or partial loss of mAb reactivity

Precise epitope mapping of mAb recognizing cancer biomarkers is substantial for the interpretation of IHC staining patterns in cancer research and to provide an accurate IHC diagnosis in clinical oncology. For instance, rationally designed anti-BRAF, anti-EGFR, or anti-p53 mAb have been generated that recognize specifically hotspot mutations in these cancer-relevant proteins, which could be highly valuable for IHC-based precision diagnosis and for novel potential precision therapies.^74–76^ In addition, the usage of mAb for high-sensitivity biomarker quantification by other methods is also dependent on their specific epitope recognition. However, it is frequent that mAb used in research, or those under standardization and validation for their routine use in the clinics, were not obtained against a rationally designed epitope, making important the definition of the recognized epitope in the context of the whole target protein. Here, we have performed a precision epitope mapping of six commercial anti-PTEN mAb suitable for IHC whose reactivity towards PTEN at a highly precision level was unknown. Short overlapping linear epitopes (6–11 amino acids length; PTEN residues 386–402) were identified at the PTEN C-terminal IDPR for all the mAb (Fig. 5). Although in some cases undisclosed PTEN C-terminal synthetic peptides were used as immunogens in the obtaining of the mAb analyzed, in some others the immunizations involved larger PTEN regions (Table 1), highlighting the immunodominance of the very C-terminal amino acid sequence of PTEN. This precludes the potential use of combinations of the tested anti-PTEN mAb for standardization of high-sensitivity sandwich immunodetection methods for PTEN quantification. In addition, most of the available commercial anti-PTEN mAb, including conjugated mAb suitable for other techniques such as flow cytometry, have been obtained by immunization with PTEN C-terminal peptides.^77^ IDPRs are expected to be exposed to the solvent and are proposed to act as adaptable sequence signals that dynamically regulate protein interactions and function.^78^ Whether this could explain the immunodominant effect of the PTEN C-terminal IDPR deserves further study. PTEN protein is extremely conserved between human and rodents, with an amino acid identity of 99–100%. The only difference between human and mouse PTEN proteins reside in the 398 residue (Thr in human; Ser in mouse), and the human PTEN variant T398S (mimicking mouse PTEN) was recognized by all the mAb analyzed.

Our precise mapping of PTEN C-terminal linear epitopes allowed us to check in silico for potential human antigens cross-reacting with the analyzed anti-PTEN mAb. We did not detect cross-reactivity with SERPINB9 and CAMK2G, two potential candidates with relevance in human cancer. Some of the mAb analyzed in our study provided IHC staining results suggestive of false positive staining, when compared with FISH results from the same samples, although we cannot completely rule out the existence in the tested samples of false negative results for *PTEN* gene absence in our FISH analysis.

Regulation of PTEN function is exerted at multiple levels, including cell type- and extracellular cue-dependent post-translational modifications.^20^ Interestingly, PTEN variants T398E and T398D (but not T398Q and T398N), which may mimic PTEN phosphorylation at Thr398 by the DNA damage-responsive ATM kinase,^73^ were not recognized by SP218 and 17.A anti-PTEN mAb. Furthermore, PTEN variants found in tumors or in the germline of patients were differentially recognized by the anti-PTEN mAb, even though these PTEN variants did not show major functional alterations. This is relevant for the interpretation of IHC results from tumor specimens using these mAb, or for PTEN quantification from human biopsies, which would be indicative of loss of PTEN protein if the samples carry those PTEN alterations. *PTEN* gene mutations generating premature termination codons are relatively abundant in tumors and in patients, and give rise to unstable truncated PTEN proteins.^51^ In addition, it has been described a PTEN-Δ splice isoform, which lacks the PTEN residues 344-403 encoded in exon 9 as a result of incorporation of intron 8 into the PTEN mRNA.^37,38^ Finally, PTEN can be cleaved by caspase-3 at residues Asp371, Asp375, and Asp384, generating catalytically-competent PTEN truncated forms with altered stability and subcellular localization, and with defective interaction with protein partners^44,53^ (Fig. 5). Our epitope mapping indicates that, regardless their functional properties, pathologic or physiologic PTEN C-terminal truncations lack the immunodominant epitopes recognized by the anti-PTEN mAb analyzed in our study. Dedicated work is required to obtain, characterize, and standardize anti-PTEN mAb recognizing epitopes at other PTEN defined regions.^79^

## Methods

### IHC and FISH

For IHC and FISH, tissue microarrays (TMA) histological sections from 81 retrospectively-obtained formalin fixed paraffin embedded (FFPE) prostate adenocarcinoma tumors and 49 retrospectively-obtained urothelial bladder carcinoma tumors were used. For IHC, samples were classified as positive or negative for staining. The antibodies and dilutions used for IHC were: 6H2.1 (1/50 in Tris/EDTA pH 9; 04-035, Merck Millipore), SP218 (1/100 in Tris/EDTA pH 9; Spring Bioscience), 17.A (Ab-4) (1/1 in Citrate pH 6.1; #MS-1601, Thermo Fisher Scientific), Y184 (1/100 in Citrate pH 6.1; ab32199, Abcam), 138G6 (1/50 in Tris/EDTA pH 9; #9559, Cell Signaling Technology), and D4.3 (1/20 in Tris/EDTA pH 9; #9188, Cell Signaling Technology). Immunostaining was performed in automated immunostainers (EnVision FLEX, Dako Autostainer Plus; Dako, Glostrup, Denmark) following routine methods. Fluorescence in situ hybridization (FISH) was performed using dual color probe containing a centromeric probe for chromosome 10 (CEN10, orange spectrum) and PTEN probe at 10q23 (PTEN, green spectrum) (Zytolight, SPEC PTEN/CEN 10 Dual Color Probe, Z-2078-200, ZytoVision, Germany). Briefly, the 5 μm TMA sections were deparaffinized, air-dried and dehydrated in gradient ethanol, followed by denaturation in 10 mM citric acid buffer for 4 min using a pressure cooker. After treatment with proteinase K during 20 min at 37 °C and washing twice in SSC Wash Buffer, probes were added and denaturation was performed at 75 °C during 10 min, followed by hybridization at 37 °C for 16 h, according to the manufacturer's directions. Slides were subsequently washed and counterstained with DAPI (Sigma-Aldrich). Stained slides were manually interpreted by fluorescence microscope, and the predominant FISH signal numbers were recorded in each tissue spot. For each case, a minimum of 50 non-overlapping interphase nuclei were evaluated. The PTEN deletion was defined as ≥15% of tumor nuclei containing one or no PTEN locus signal and two CEP10 signals. This study is approved by the corresponding institutional Ethical Committees (CEIC E16/51 and FIU-AEU-2016).

### Cell lines, cell culture, and transfections

Simian kidney COS-7 cells and human breast adenocarcinoma MCF7 cells were grown in DMEM containing high glucose supplemented with 5 and 10% heat-inactivated fetal bovine serum (FBS), respectively, 1 mM l-glutamine, 100 U/ml penicillin, and 0.1 mg/ml streptomycin. Human prostate adenocarcinoma LNCaP cells and human renal carcinoma Caki-1 cells were grown in RPMI, containing 10% heat-inactivated FBS, 1 mM l-glutamine, 100 U/ml penicillin, and 0.1 mg/ml streptomycin. Human glioblastoma U87MG cells were grown in DMEM containing high glucose supplemented with 10% heat-inactivated FBS, 1 mM l-glutamine, 1 mM sodium pyruvate, 1% nonessential amino acids, 100 U/ml penicillin, and 0.1 mg/ml streptomycin. Cells were grown at 37 °C and 5% CO_2_. Cells were transfected by the DEAE-dextran method (COS-7 cells) or using Lipofectamine (Thermo Fisher Scientific, USA) (MCF7, LNCaP, Caki-1, and U87MG cells), and processed after 48 h.

### Plasmids and mutagenesis

pRK5 PTEN, pRK5 GST-PTEN, pRK5 Flag-PTEN, and pSG5 AKT1 plasmids have been previously described.^52,55,57^ pRK5 PTEN-L (residues 1-576) was generated by subcloning from the plasmid pGEX 6P1 PTEN-L (provided by N. Leslie) and adding the first 20 N-terminal PTEN-L amino acids by PCR. pRK5 PTEN-Δ (residues 1-343-Ser), as well as the PTEN and GST-PTEN amino acid substitution variants, were made from the plasmids pRK5 PTEN and pRK5 GST-PTEN by PCR oligonucleotide site-directed mutagenesis, as described.^80^ pCDNA3.1 SERPINB9-Flag (NP_004146.1) and pReceiver CAMK2G-Flag (NP_751911.1) plasmids were purchased from GenScript and GeneCopoeia, respectively. All mutations were confirmed by restriction digestion and DNA sequencing (Fig. S2). Nucleotide and amino acid numbering for PTEN variants correspond to reference sequences from accession numbers NM_000314.4 and NP_000305.3, respectively.

### Immunoblotting

Whole-cell protein extracts from cell lines were prepared by cell lysis in ice-cold M-PER^TM^ lysis buffer (Thermo Fisher Scientific) supplemented with PhosSTOP phosphatase inhibitor and cOmplete protease inhibitor cocktails (Roche, Switzerland), followed by centrifugation at 15,200 × *g* for 10 min and collection of the supernatant. Cell lysates were subjected to sodium dodecyl sulfate-polyacrylamide gel electrophoresis (10% SDS-PAGE). Proteins (50–100 μg) were resolved under reducing conditions and transferred to PVDF membranes (Immobilon-FL, Millipore). Immunoblotting was performed using the anti-PTEN antibodies indicated above, at the following dilutions: 6H2.1 (1/1000), SP218 (1:400), 17.A (1:1), Y184 (1/5000), 138G6 (1/1000), D4.3 (1/1000). Other antibodies used in immunoblotting were: polyclonal anti-GST,^57^ polyclonal anti-PTEN N-terminal,^52^ anti-phospho-Ser473-AKT and anti-AKT (both from Cell Signaling Technologies), anti-GAPDH (Santa Cruz Biotechnology), and anti-Flag (Sigma-Aldrich). Secondary antibodies conjugated with fluorochrome were anti-rabbit or anti-mouse IgG-IRDyeR 800CW (or IgG-Alexa FluoR 680) (LI-COR Biosciences). For determination of phospho-AKT content and the relative amount of PTEN protein detected by the different antibodies, bands were quantified using an Image studioTM software with Odyssey® CLx Imaging System (LI-COR, USA). For all comparative results shown, blots derive from the same experiment and were processed in parallel.

### Immunofluorescence

PTEN subcellular distribution in COS-7 cells was determined by immunofluorescence using mouse monoclonal anti-PTEN 425.A^79^ and fluorescein-conjugated anti-mouse antibody (Thermo Fisher Scientific). For quantitation of PTEN subcellular distribution, at least 50 positive cells were scored for each experiment. Cells were rated as nuclear staining (N), cytoplasmic staining (C), or staining within both the nucleus and the cytoplasm (N/C). Nuclei were identified by DAPI staining.

### Reporting Summary

Further information on experimental design is available in the Nature Research Reporting Summary linked to this article.

## Supplementary information

Table S1, Fig. S1, Fig. S2, uncropped gels

Reporting Summary

## Data Availability

The authors declare that all data supporting the findings of this study are available within the paper and its supplementary information files.

## Supplementary information

**Supplementary Information** accompanies the paper on the *npj Precision Oncology* website (10.1038/s41698-019-0083-4).
