# Supplementary material for: Precise definition of PTEN C-terminal epitopes and its implications in clinical oncology
Source: NPJ Precis Oncol. 2019 Apr 15;3:11. doi: 10.1038/s41698-019-0083-4 (PMC6465295; doi:10.1038/s41698-019-0083-4)
Supplement: Supplementary file 1 — Table S1, Fig. S1, Fig. S2, uncropped gels [file 41698_2019_83_MOESM1_ESM.pdf]

Table S1. Comparative IHC and FISH analysis of anti-PTEN mAb using a panel of FFPE urothelial bladder carcinomas

			FISH-		*FISH+*		*FISH++*[1]	
mAb	neg/pos[2]	%neg	neg	pos	neg	pos	neg	pos
**6H2.1**	25/24	51	0	0	2	1	23	23
**SP218**	28/21	57.1	0	0	2	1	26	20
**17.A**	29/20	59.2	0	0	2	1	27	19
**Y184**	12/37	24.5	0	0	0	3	12	34
**138G6**	27/22	55.1	0	0	2	1	25	21
**D4.3**	33/16	67.3	0	0	2	1	31	15

[1] -, no PTEN signal; +, 1 PTEN signal; ++, 2 PTEN signals
[2] -, number of negative/positive samples for IHC staining

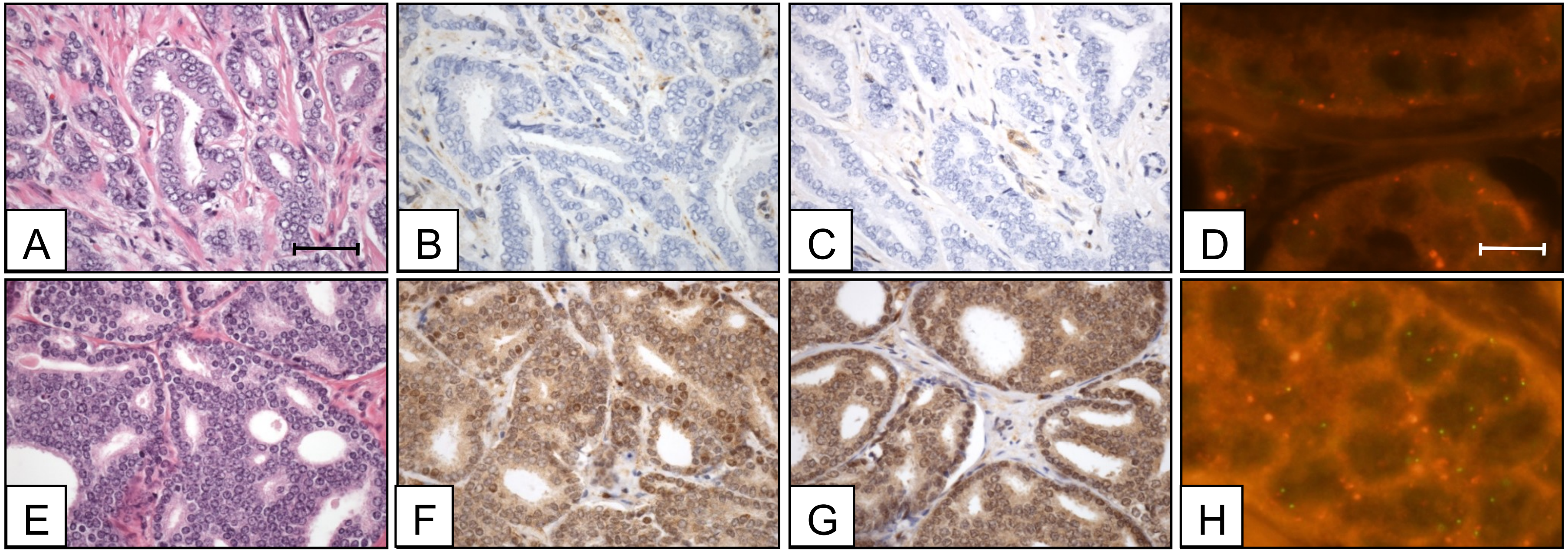

Figure S1. IHC staining with the anti-PTEN 6H2.1 and SP218 mAb, and *PTEN* FISH analysis, of selected prostate tumor samples. **A, B, C, D)** Prostate adenocarcinoma with *PTEN* gene biallelic loss and PTEN protein loss, Gleason index 3+3. **E, F, G, H)** Prostate adenocarcinoma with *PTEN* gene and PTEN protein preservation, Gleason index 4+4. **A and E**, Histological hematoxylin-eosin staining; **B and F**, IHC with 6H2 mAb; **C and G**, IHC with SP218 mAb; **D and H**, FISH analysis. A, B, C, E, F, and G, magnification X200; in A, a representative scale bar = 100 μm is shown. D and H, magnification X1000; in D, a representative scale bar = 20 μm is shown.

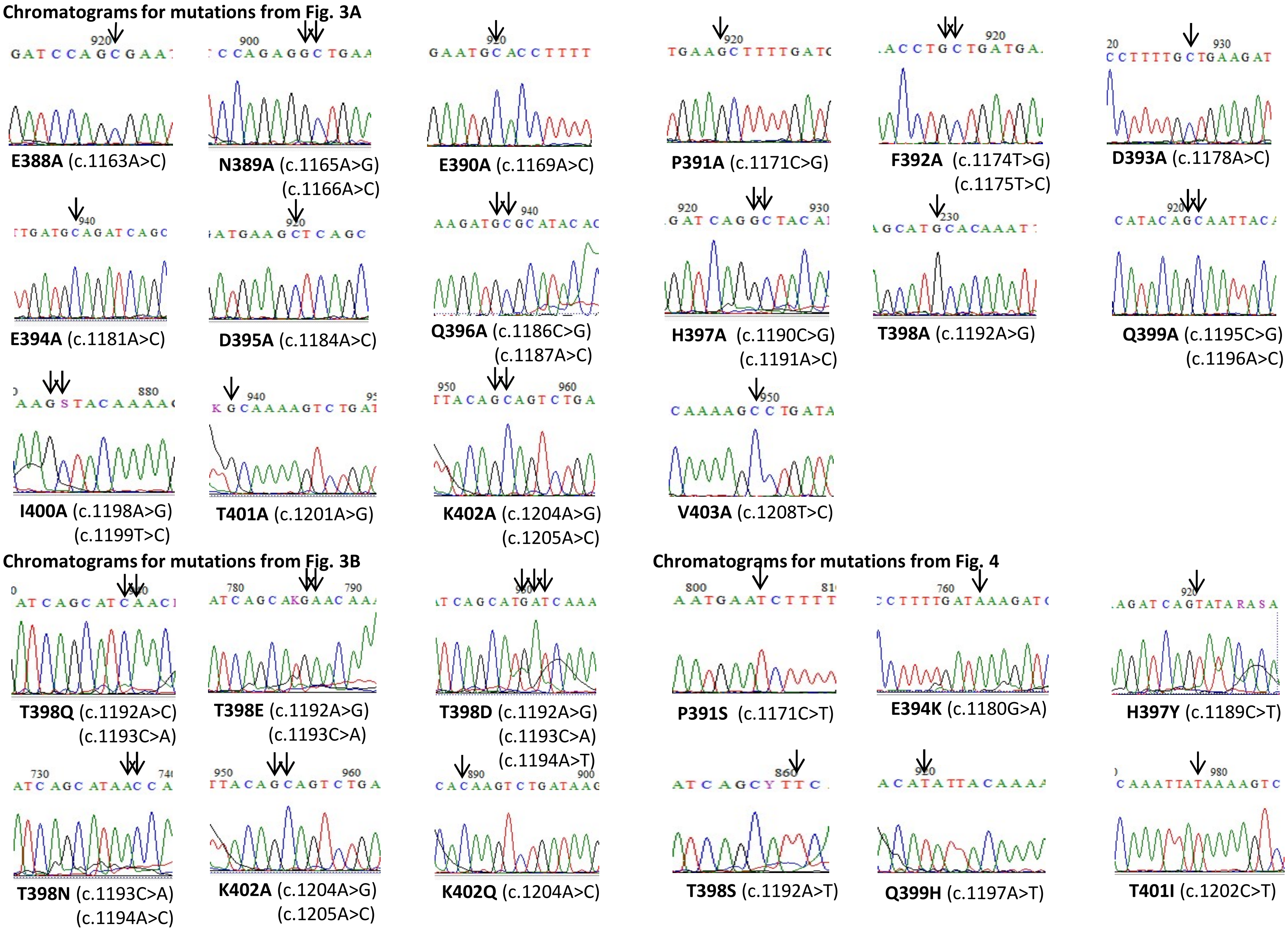

Figure S2. DNA sequencing analysis of PTEN amino acid substitution variants. Nucleotide sequence ABI chromatograms from the PTEN amino acid substitution variants used in our study are shown. Nucleotide numbering corresponds to accession NM_000314.4. Variant nomenclature is according to HGVS.

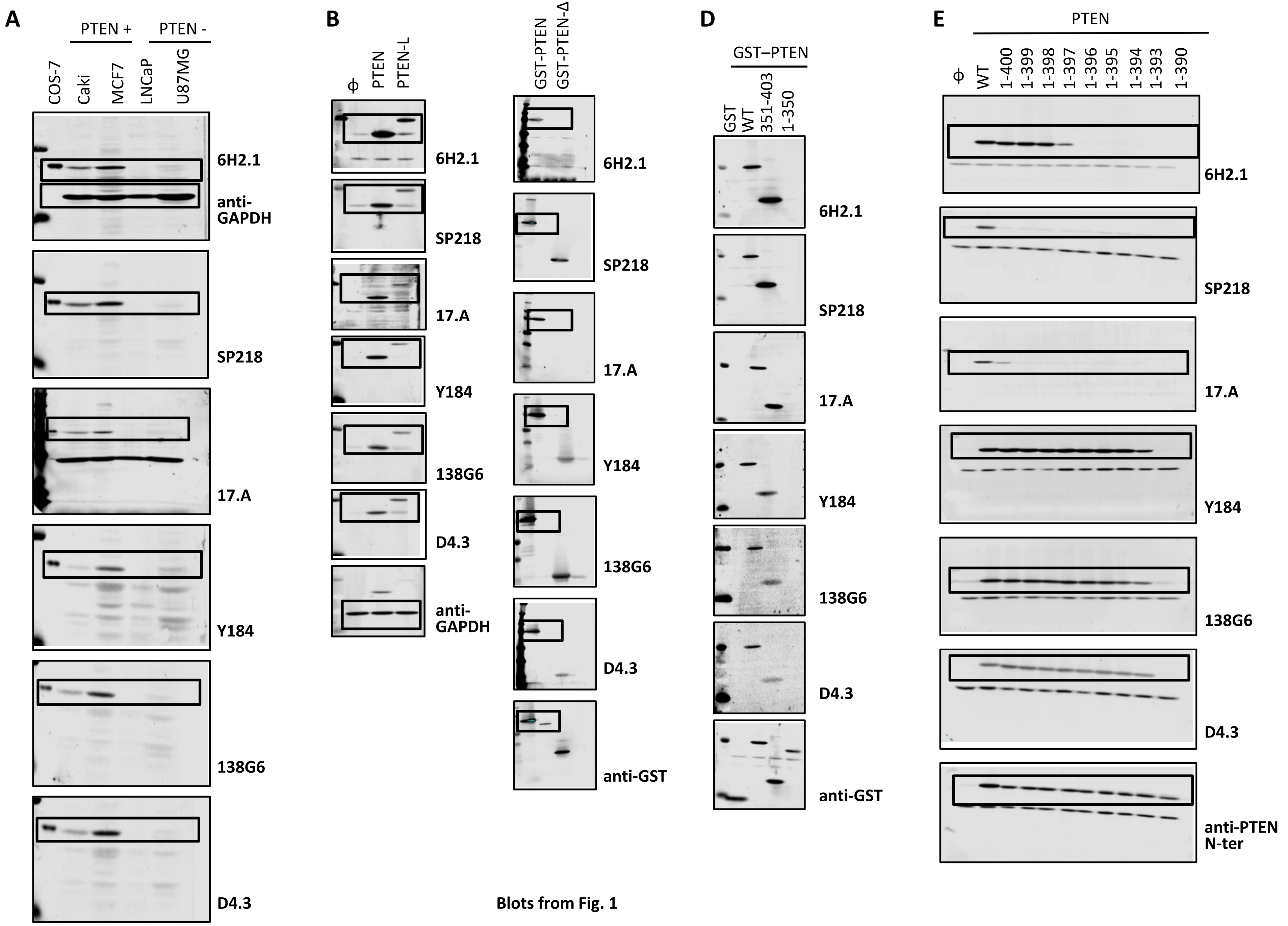
A
PTEN +
PTEN -
COS-7
Caki
MCF7
LNCaP
U87MG
6H2.1
anti-GAPDH
SP218
17.A
Y184
138G6
D4.3
B
ϕ
PTEN
PTEN-L
6H2.1
SP218
17.A
Y184
138G6
D4.3
anti-GAPDH
GST-PTEN
GST-PTEN-Δ
6H2.1
SP218
17.A
Y184
138G6
D4.3
anti-GST
D
GST–PTEN
GST
WT
351-403
1-350
6H2.1
SP218
17.A
Y184
138G6
D4.3
anti-GST
E
PTEN
ϕ
WT
1-400
1-399
1-398
1-397
1-396
1-395
1-394
1-393
1-390
6H2.1
SP218
17.A
Y184
138G6
D4.3
anti-PTEN N-ter
Blots from Fig. 1

Fig.1.

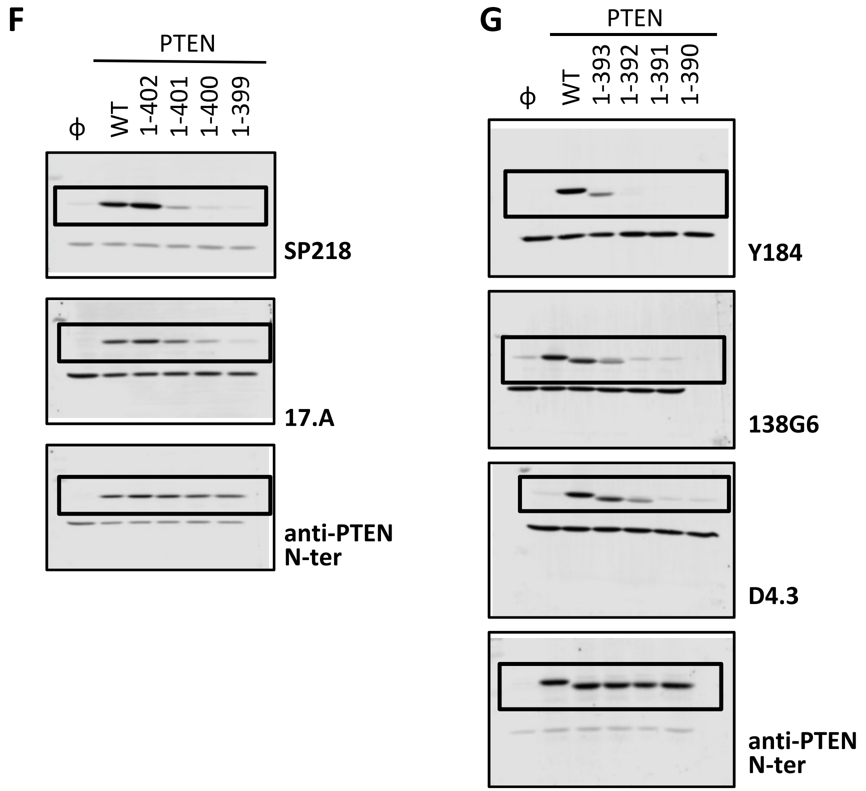

Blots from Fig. 1

Fig.1.

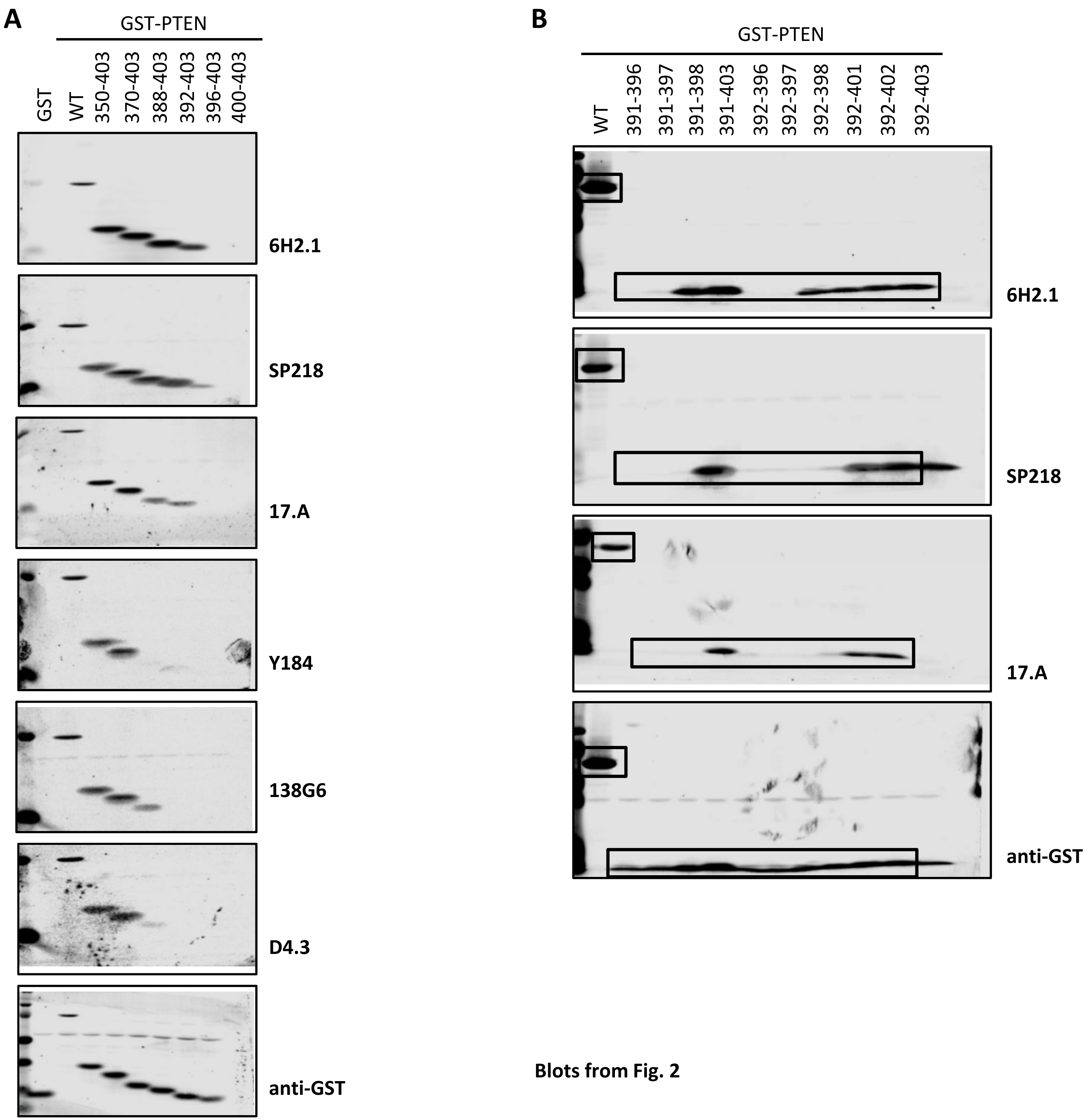

Blots from Fig. 2

Fig.2.

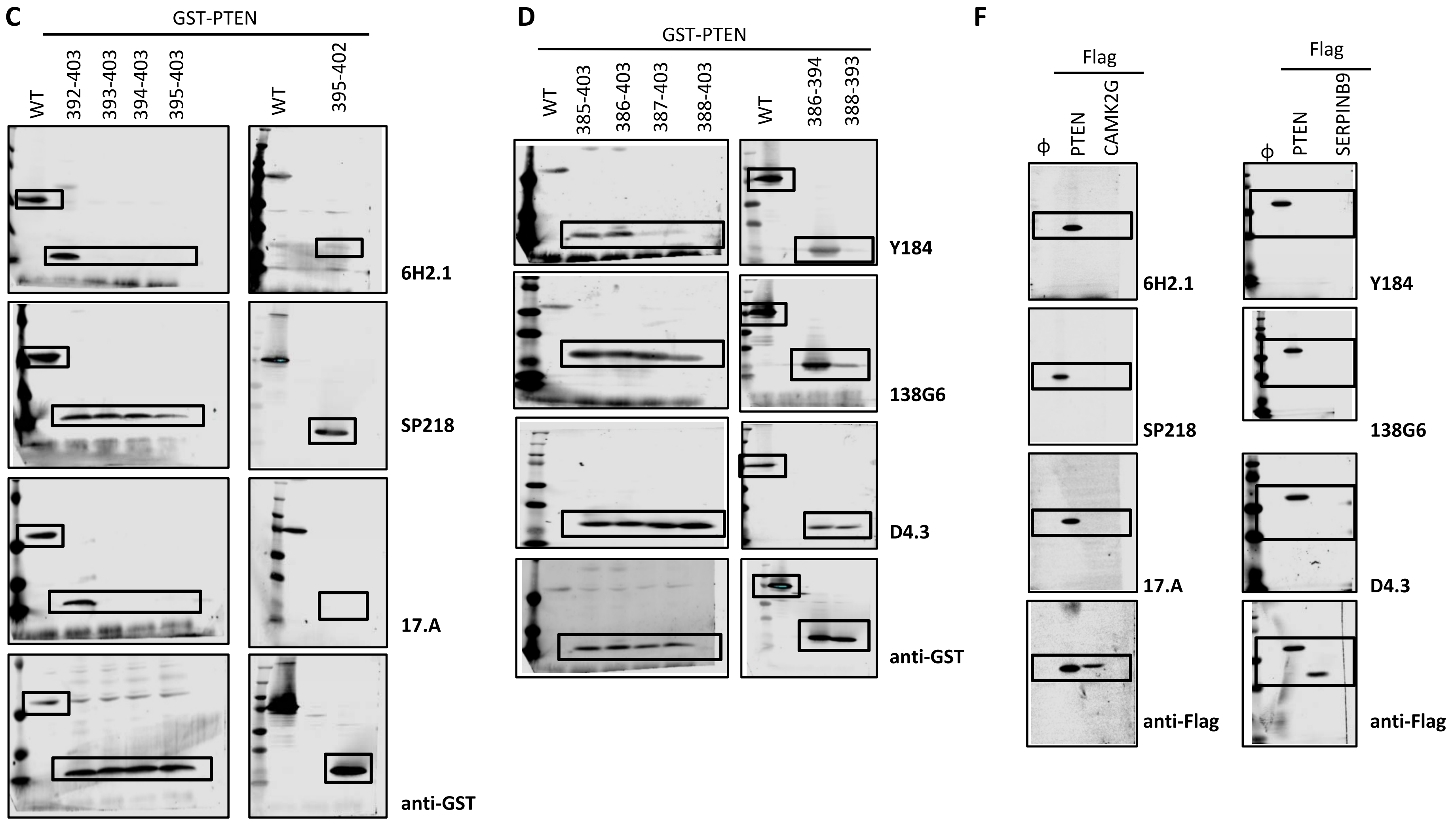

Blots from Fig. 2

Fig.2.

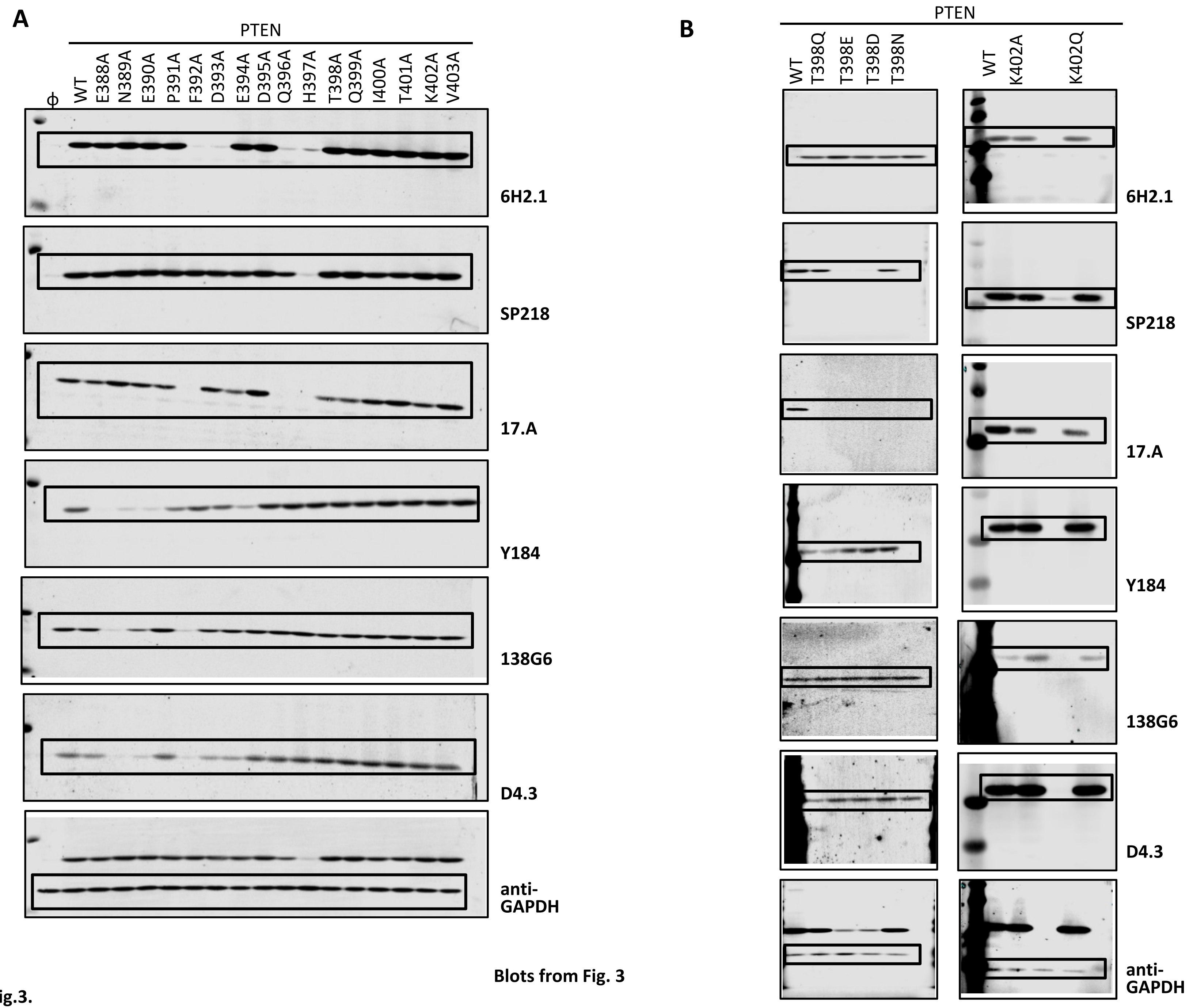

Blots from Fig. 3

Fig.3.
